# Development of an Autochthonous Microbial Consortium for Enhanced Bioremediation of PAH-Contaminated Soil

**DOI:** 10.3390/ijms222413469

**Published:** 2021-12-15

**Authors:** Marta Roszak, Joanna Jabłońska, Xymena Stachurska, Kamila Dubrowska, Justyna Kajdanowicz, Marta Gołębiewska, Anna Kiepas-Kokot, Beata Osińska, Adrian Augustyniak, Jolanta Karakulska

**Affiliations:** 1 Department of Microbiology and Biotechnology, Faculty of Biotechnology and Animal Husbandry, West Pomeranian University of Technology in Szczecin, Al. Piastów 45, 70-311 Szczecin, Poland; marta.roszak@pum.edu.pl (M.R.); joanna_jablonska@zut.edu.pl (J.J.); xymena.stachurska@zut.edu.pl (X.S.); dk37456@zut.edu.pl (K.D.); j.kajdanowicz95@gmail.com (J.K.); marta-golebiewska@zut.edu.pl (M.G.); jolanta.karakulska@zut.edu.pl (J.K.); 2 Department of Laboratory Medicine, Pomeranian Medical University in Szczecin, Al. Powstańców Wielkopolskich 72, 70-111 Szczecin, Poland; 3 Department of Chemical and Process Engineering, Faculty of Chemical Technology and Engineering, West Pomeranian University of Technology in Szczecin, Al. Piastów 42, 71-065 Szczecin, Poland; 4 Department of Environmental Management, Faculty of Environmental Management and Agriculture, West Pomeranian University of Technology in Szczecin, ul. Słowackiego 17, 71-434 Szczecin, Poland; anna.kiepas-kokot@zut.edu.pl; 5 Research Institute of Animal Production PIB Kołbacz Sp. z o.o., Warcisława Street 1, 74-106 Kołbacz, Poland; beata.osinska@kolbacz.pl; 6 Chair of Building Materials and Construction Chemistry, Technische Universität Berlin, Gustav-Meyer-Allee 25, 13355 Berlin, Germany

**Keywords:** applied microbiology, autochthonic microorganisms, bioremediation, polycyclic aromatic hydrocarbons, soil pollution

## Abstract

The main objectives of this study were to isolate bacteria from soil chronically contaminated with polycyclic aromatic hydrocarbons (PAHs), develop an autochthonous microbial consortium, and evaluate its ability to degrade PAHs in their native contaminated soil. Strains with the best bioremediation potential were selected during the multi-stage isolation process. Moreover, to choose bacteria with the highest bioremediation potential, the presence of PAH-degrading genes (*pah*E) was confirmed and the following tests were performed: tolerance to heavy metals, antagonistic behavior, phytotoxicity, and antimicrobial susceptibility. In vitro degradation of hydrocarbons led to the reduction of the total PAH content by 93.5% after the first day of incubation and by 99.22% after the eighth day. Bioremediation experiment conducted in situ in the contaminated area resulted in the average reduction of the total PAH concentration by 33.3% after 5 months and by over 72% after 13 months, compared to the concentration recorded before the intervention. Therefore, this study implicates that the development of an autochthonous microbial consortium isolated from long-term PAH-contaminated soil has the potential to enhance the bioremediation process.

## 1. Introduction

Polycyclic aromatic hydrocarbons (PAHs) are made up of two or more fused aromatic rings and are ubiquitous organic contaminants in the environment [1,2]. Being lipid soluble and quickly absorbed by mammals, with established teratogenic, mutagenic, and carcinogenic effects, 28 of these non-polar substances have been listed as priority pollutants by both the US Environmental Protection Agency (EPA) and European Union (EU) [1,2,3].

While PAHs can occur naturally, they mostly originate from anthropogenic processes. The level of PAHs in soils in urban areas is approximately 2–10 times higher than in rural areas [1]. Many former industrial sites occur near city centers and have high land value, therefore there is a need to purify the soil to meet development requirements and increase the attractiveness of those sites [4]. PAHs are highly hydrophobic, immobile, and stable chemical structures, which can be rapidly absorbed by soil particles which results in their persistence in the environment and difficulties in removing them [1,5]. The presence of PAHs in soils exerts pressure on the microbial community that suffers a reduction in soil microbial richness and also a reduction in soil functionality [6,7]. Moreover, it has been shown that they can be up-taken by crops. A study on vegetables, such as lettuce, potato, and carrot, grown in soils contaminated by PAHs showed elevated levels of several trace elements and PAHs. Vegetables that accumulate part of the dangerous substances from the soil pose a danger for consumers and are not authorized for consumption by humans and animals, which increases cost in the agriculture sector [8].

The technologies to eliminate PAHs from the natural environment include solvent extraction, chemical oxidation, photocatalytic degradation, electrokinetic remediation, thermal treatment, incineration, soil washing, bioremediation, phytoremediation, bioaugmentation, biostimulation, composting/biopiles, and bioreactors. However, it should be emphasized that, in many cases, a combination of these techniques may be the most effective, especially when the soil is chronically contaminated [3,9,10]. The biological treatment of contaminated soil (bioremediation) is defined as a more financially affordable and adaptable option than physicochemical treatments and provides strong advantages, such as the possibility to degrade pollutants with high efficiency at lower treatment cost, greater safety, and lower soil disturbance [10]. One of the bioremediation processes is bioaugmentation, which involves the addition of external microbial strains (indigenous or exogenous)**,** found in aqueous environments, soils, or sediments, to co-metabolically degrade PAHs [3,11,12]. Bioaugmentation has been considered a valuable tool for increasing the rate and extent of the biodegradation of target toxic molecules. Moreover, when using bioaugmentation, it seems more practical to use autochthonous (indigenous to the site) microorganisms because of environmental factors influencing the effectiveness of bioaugmentation [13]. Under the influence of the environmental pressure caused by PAHs, microorganisms adapt to these unfavorable living conditions, often unveiling a bioremediation potential. Organic pollutants act as a C source for environmentally adapted organisms, which through co-metabolism and synergism are able to remove the contaminant. Quite frequently, bioremediation releases intermediates of degradation pathways which could be less toxic to the environment [14]. Therefore, these autochthonous microorganisms, if isolated, cultivated, and introduced back to the soil, can efficiently remove PAHs without the necessity to implement any foreign strains [10]. Microorganisms used for bioaugmentation are often used in the form of microbial consortia, which, in general, are more efficient in removing PAHs from contaminated sites because of a diversity of enzymatic activities and synergistic effects, increasing biodegradation performance [15]. Microbial consortia may consist of bacteria, ligninolytic fungi, algae, or mixed cultures [16,17], and could consist of between two and fifty isolates, with five on average [3,18,19,20,21,22,23]. The choice of microorganisms for the consortium development requires selection, including confirmation of the presence of genes responsible for the production of enzymes that break down PAHs. Culture-independent methods, especially genetic methods, are applied to identify diversity and microbial groups associated to PAH degradation in sites. The most widely used approach is based on 16S rRNA gene-PCR amplification which is used to associate the known metabolic profile of the taxon with bioremediation potential [24]. Apart from that, PAH-degrading genes such as *nahAc, phnAc, nagAc, pahAc,* and *pahE,* etc., can be detected to directly associate the bioremediation potential with a given strain [17,25].

Even though autochthonous bioaugmentation has great potential to reduce PAH concentrations in the environment, and this strategy was previously discussed in the literature, a limited number of reports have been published on autochthonous bioaugmentation in situ [13]. In this study, we hypothesized that the contaminated soil can be used to obtain an autochthonous microbial consortium for the efficient bioaugmentation of soil contaminated with phenol and PAHs from roofing tar production, that can then be applied on the surface of the soil and into deeper layers by boreholes to assist the bioremediation process. PAH pollution in our area of interest was monitored throughout the years (2015–2018) and the concentration of PAHs was not significantly lowered during that time. For that reason, our study aimed at: (a) isolating bacteria from an aged, PAH-contaminated soil, (b) developing an autochthonous microbial consortium, and (c) evaluating its ability to reduce the content of selected PAHs in laboratory and native environments.

## 2. Results

### 2.1. Bacteria Isolation and Identification

In total, 44 strains were isolated from contaminated soil and subjected to further selection. After the spot test assay, 21 strains were selected based on their ability to grow on the minimal medium enriched with PAHs. The second test, conducted in the liquid medium, led to the selection of the final 14 strains, characterized as bacteria with a potential to decompose PAHs and phenol. The analysis of the 16S rRNA gene indicated that these 14 isolates represented seven genera: *Achromobacter* sp. (strain 20), *Arthrobacter* sp. (strains 26, 27, 29), *Microbacterium* sp. (strain 11), *Pseudomonas* sp. (strains 17, 36, 38, 41), *Cupriavidus* sp. (strain 22), *Rhodococcus* sp. (strains 24, 40, 44)*,* and *Streptomyces* sp. (strain 19). Isolates were deposited in the GenBank database under the following accession numbers: MT755842-MT755855 (forward) and MT755859-MT755872 (reverse). The detailed characterization of isolates is presented in the Supplementary Materials (Tables S2–S7, Figures S1 and S2).

### 2.2. Detection of PAH-Degradation-Related Gene

Isolated strains differed concerning PAH-degradation gene presence. The assay resulted in at least one product for every tested strain (strains 11, 19, 20), however, most of them were characterized with two or more products. The *pahE1* product was present in 9 strains, *pahE2* in 4 strains, *pahE3* in 11 strains, and *pahE4* in 12 strains. The detailed results are presented in Table 1.

### 2.3. Effectiveness of In Vitro Hydrocarbon Degradation

After the first day of in vitro tests, the amount of every tested substance was significantly lower than the initial calculated values (100%), both in the tested and control samples, shown in Table 2. The absolute values are shown in the Supplementary Materials (Table S8). The content of the total PAHs after the first day decreased from 8300 µg/L to 1600 µg/L in the control sample and 650 µg/L in the tested one. After the eighth day of incubation, the content of the PAHs decreased and reached 930 µg/L and 78 µg/L for the control and tested sample, respectively. Although the reduction of the PAHs was also observed in the control sample, it was greater in the sample containing the microbiological consortium. After the first day, the reductions of phenanthrene, anthracene, fluoranthene, chrysene, and the total PAHs in the tested sample were at least twice as big as in the control samples. At the end of the experiment, the reduction values of the total PAHs between the control and tested samples differed approximately tenfold. The mixture of PAHs, after treatment with the bacterial consortium, led to the almost complete removal of the selected PAHs, i.e., less than 2% of each compound was detected in comparison to the initial concentration.

### 2.4. In Situ Application of Microbiological Consortium

The permissible concentrations of PAHs were exceeded in the studied soil, i.e., over 250 mg/kg for the total of PAHs and 50 mg/kg for each hydrocarbon. In contrast, the phenol content was lower than identified as contamination under the Regulation of the Minister of the Environment of 9 September 2002 on soil quality standards and ground quality standards (*Journal of Laws of the Republic of Poland* 2002 No. 165, item 1359). The bioremediation effect on the remediation area was assessed 5 and 13 months after the application of the consortium onto the polluted area (Table 3 and Table 4). The total concentration of PAHs decreased at Spot 1 from 2635.0 mg/kg (100%) (May 2019) to 2344.0 mg/kg (88.9%) in October 2019 and then to 977.4 mg/kg (37.1%) in June 2020. In spite of the fluctuation of particular compound concentrations, the final values of each hydrocarbon were lower than at the beginning. At Spot 2, phenol, phenanthrene, anthracene, and chrysene reduction were obtained in October 2019 (19.7%, 84.0%, 82.6%, and 59.9% of the initial value, respectively). However, other compounds and the total PAH concentrations increased (naphthalene 119.4%, fluoranthene 285.7%; total PAHs 174.7% of initial value). The ecological effect was obtained only later, in June 2020, when the total PAHs decreased by more than a half (48.21% of the initial value). Spot 3 was significantly purified from hydrocarbons in October 2019 (7% of the initial value), although in June 2020 an increase in fluoranthene concentration to 186.4% of the initial value had influenced the final result. The mean reduction value of every hydrocarbon decreased after 13 months from the applications of the bacterial consortium in comparison to the results from May 2019 (with two exceptions: phenanthrene (Spot 2) and fluoranthene (Spot 3)). The most impressive change was observed in the phenol index at all spots. Other compounds′ reduction varied depending on the spot.

## 3. Discussion

In this work, the selection process has led to the formulation of the microbiological consortium containing 14 isolates that have been tested in vitro to confirm their biotechnological potential. Detailed data for this part (apart from PAH degradation that is the main objective in this study) is presented in the Supplementary Materials and discussed below.

The primary group included 21 strains, however, seven of them were rejected due to a lack of efficient growth in the liquid cultures. Perhaps these bacteria were oligotrophs that could utilize agar as a sole carbon source, or the environment of the liquid medium was not suitable for their growth [26]. Another possibility is that they could be more sensitive to the contamination in the liquid medium, because of the increased availability of substances in this environment [27]. The isolates (*Arthrobacter* sp., *Pseudomonas* sp., *Rhodococcus* sp., and *Streptomyces* sp.) obtained in our study belong to taxa previously associated with biodegradation [11,28]. However, other genera often implemented in the bioremediation process, such as *Alcaligenes* sp., *Burkholderia* sp., *Comamonas* sp., *Flavobacterium* sp., *Micrococcus* sp., *Mycobacterium* sp., *Pasteurella* sp., *Sphingomonas* sp., and *Vibrio* sp., were not found in our soil samples [11,28]. The taxonomic affiliation of the isolates has been confirmed with the analysis of the 16S rDNA sequence. Furthermore, the obtained phylogenetic tree, along with biochemical characteristics of the isolates, indicated that the isolates belonging to the same species were different strains. These results are shown in the Supplementary Materials (Tables S2 and S3). 

Heavy metal contamination of soil was tested before the research, and concentrations of these pollutants (Supplementary Materials, Table S1) were not overdrawing the values included in the Regulation of the Minister of the Environment of 9 September 2002 on soil quality standards and ground quality standards (*Journal of Laws of the Republic of Poland* 2002 No. 165, item 1359). Nevertheless, the tolerance to heavy metals (Supplementary Materials, Table S4) was tested due to the frequent co-existence of xenobiotics and heavy metals in soils contaminated with PAHs [29,30,31]. Concentrations of heavy metals chosen for the tolerance experiment ranged from 25 to 0.20 mM (for Zn, Cu, Cd), 12.5 to 0.10 mM (Co and Ni), 7.5 to 0.06 mM (Pb), and 3.75 to 0.03 mM (Hg), and were similar to the concentrations used by other groups [20,21,22,23]. Strain no. 17 was reported with the maximum tolerable concentration (MTC) of 7.5 mM for Pb which is greater than values obtained by Pages et al. [32] and Bhojiya and Joshi [33], where MTC was 5 mM and 0.75 mM, respectively. Isolates no. 36 and 38 presented tolerance to 6.25 mM of Cu, as opposed to 5 and 1 mM, respectively, obtained by other groups. The results of MTCs for Ni ranged from 0.39 to 1.56 mM and are slightly lower than that presented by Bhojiya and Joshi [33]—2 mM— and significantly lower than that reported by Pages et al. [32]—10 mM. However, the examined tolerance to Co was between the value obtained by Pages et al. [32]—0.1 mM— and Bhojiya and Joshi [33]—7 mM. The tolerance to Cd salts varied among the strains and ranged from 0.39 to 6.25 mM with only two susceptible bacteria in the whole group. No resistance was detected to mercury, which was proved to be the most toxic [34,35]. Pseudomonads obtained the highest MTC values in most cases, which confirms results found in the literature [33,36,37,38]. Furthermore, the increased tolerance to heavy metals was expected in the prepared consortium, as genera other than *Pseudomonas* sp., such as *Arthrobacter* sp., *Streptomyces* sp., *Microbacterium* sp. or *Rhodococcus* sp., have been previously mentioned as bacteria tolerant to high concentrations of heavy metals, making the consortium less vulnerable to the negative effects associated with heavy metal contamination that often co-occurs with PAH contamination [34,39,40,41,42,43]. Obtained results showed the elevated tolerance of bacteria selected for the consortium to relatively high concentrations of heavy metals, which strengthened their potential to be used in the bioremediation of contaminated soil (see Supplementary Materials, Table S4).

Agricultural, urban, and pristine soils are suspected to be an important reservoir of antibiotic resistance genes, and a potential source for antibiotic resistance in pathogenic bacteria infecting animals and humans [44,45,46,47]. It is likely that anthropogenic pollutants, such as heavy metals and PAHs, may contribute to increased antibiotic resistance in bacteria, either via co-resistance or cross-resistance [48,49,50,51]. Chen et al. [50] and Wang et al. [49] discovered that the presence of PAHs can lead to the dissemination of antibiotic resistant genes in soil bacteria. In addition, Maurya et al. [52] pointed out the connection between PAHs and genes encoding efflux pumps, that may increase antibiotic resistance in environmental bacteria. Even though we found an increased antibiotic resistance (particularly in pseudomonads) in the selected group, the 16S analysis and general growth patterns indicated that none of the detected bacteria belonged to species with a high clinical significance [53,54] (see Supplementary Materials, Tables S6 and S7). The BLAST engine did not suggest a strong association with clinically relevant species such as *Pseudomonas aeruginosa* or *Achromobacter xylosoxidans*, whereas streptomycetes are generally known for their increased resistance to antibiotics [55]. For these reasons, all 14 strains have been included in the final consortium.

Most of the consortium strains (9 of 14) did not show any observable antagonism against other strains in the laboratory conditions, while the minority expressed only slight effects. Therefore, all 14 strains were admitted to the final consortium (Supplementary Materials, Figure S1 and Table S5). According to the literature, the antagonistic activity of *Pseudomonas* spp. towards bacteria and fungi does not advise against including them in a bioremediation consortium [56]. Similarly, the antagonistic effect of the *Streptomyces* sp. against *Arthrobacter* spp. observed during the study is a known phenomenon in the literature [57]. Little is known about the antagonistic interactions between *Rhodococcus* spp. and *Pseudomonas* spp., also observed in this study. However, *Rhodococcus* spp. was previously reported to inhibit bacterial and fungal growth [58,59]. Moreover, a high bioremediation potential of *Rhodococcus* spp. indicated in the literature [60,61,62] outweighs its weak inhibitory activity against *Pseudomonas* spp., allowing their inclusion in the consortium. 

To avoid introducing an excessive number of phytotoxic bacteria to the soil, the phytotoxicity assay was performed. Garden cress (*Lepidium sativum*) was chosen due to its rapid germination and growth [63]. In the presented research, seven strains out of fourteen affected the growth of neither shoots nor roots in *L. sativum* seeds in comparison to the control samples. Despite some bacterial strains causing the significantly shorter length of the shoots or roots, the tested seedlings grew, and further plant development was possible (Supplementary Materials, Figure S2). Our consortium was proven not to harm the used plant model; however, special attention should be paid to the studies of the interactions between microorganisms used in bioremediation, and plants, to maximize their potential and avoid introducing phytopathogens into the soil [64].

Next to the basic culture methods, a real-time PCR was conducted to evaluate the presence of genes associated with PAH degradation. The obtained results after the isolation process confirmed the high potential of the investigated bacteria to degrade PAHs. In this work, gene *pahE* was selected as a biomarker for PAH-degrading bacteria. *PahE* encodes hydratase-aldolase which converts analogs of trans-o-hydroxybenzylidenepyruvate (tHBPA) to aldehydes and pyruvic acid [65]. To specifically amplify partial stretches of the *pahE* gene sequence from different clads we used four primer sets (PahE1, PahE2, PahE3, and PahE4), as was proposed by Liang et al. [25]. The *pahE1* gene was designed for *Betaproteobacteria* and *Gammaproteobacteria*, *pahE2* for *Alfaproteobacteria*, *pahE3* for *Rhodococcus,* and *pahE4* for *Mycobacteria*. However, our results indicated that most of the strains demonstrated not only genes specific for their clad but also others. 

Soils contaminated with PAHs for a long time can be a good source of preselected microorganisms with biotechnological potential. Bacteria living in stress conditions create adaptational mechanisms that allow them to survive [66]. Since bacteria often use syntrophic relations, the advisable approach is to create a mixture of microorganisms with varying degradation capabilities [67,68]. The enzymatic activity of the primary metabolism for the consortium confirmed that the selected group expressed a broad range of enzymes responsible for amylolytic, lipolytic, and proteolytic activities. Such enzymatic diversity increases the chance of survival of the consortium members in the natural environment and confirms that the autochthonic biota in the studied area expresses relatively high adaptability to the changing environmental conditions. Broad enzymatic capabilities also may be useful in the biodegradation of other contaminants, e.g., parabens or pesticides [69,70]. 

The only enzymatic activity that was not detected was α-fucosidase, responsible for the hydrolysis of the α-fucoside to fucose and alcohol (Supplementary Materials, Table S3). Fucose, a substrate for this enzyme, has relatively low abundance in the biosphere, which may explain why none of the isolates expressed the α-fucosidase activity [71]. 

As presented in the previous paragraphs, we have successfully collected and mixed a microbiological consortium with the potential to degrade PAHs and survive in a harsh, contaminated environment. In the next stage of the project, this consortium was applied in situ and the contamination was monitored.

The recorded contamination was significantly reduced during the studies. However, it should be emphasized that obtained values were characterized with relatively high deviation. Nevertheless, this comes with an agreement with studies focusing on PAH soil pollution, where the wide range of PAH content in soils, together with their high variability, was also noted [72,73,74]. The development of a consortium consisting of autochthonous bacteria and its successful application in situ was not often described in the literature. However, this objective was achieved in this study in laboratory conditions, much like those reported by other authors [25], and in situ.

During the experiment, the hydrocarbon content of soil may have been influenced by the special conditions of the study area. The increase in the average fluoranthene content observed after 5 months of the experiment indicates that the variability was multidirectional. The influence of terrain on the presence, distribution, and migration of PAHs in soil was also confirmed by other authors [75,76]. Moreover, Li et al. [77] stated that the concentration and pattern of soil PAHs are not only related to their input from polluted sources but also associated to soil properties, which influence their loss processes, including sorption and desorption, transportation, leaching, volatilization, as well as biological intake and decomposition. Despite the possible co-occurrence of these factors, our bioremediation study showed that the content of PAHs and phenol index was reduced, and the average content of all hydrocarbons tested during the 13-month-long experiment decreased (with two exceptions (Table 4)). The recorded results suggest that the potential effect of our microbiological consortium cannot be excluded. For example, the reduction in phenol index by 92.09% may suggest the increased degradation of aromatic compounds, which could be accounted for the biological activity [78]. Although the reduction of PAHs was meaningful, the desirable ecological effect according to the Regulation of the Minister of the Environment of 9 September 2002 on soil quality standards and ground quality standards (*Journal of Laws of the Republic of Poland* 2002 No. 165, item 1359) (individual hydrocarbons 50 mg/kg; total PAH 250 mg/kg) was not reached (excluding phenol index at Spot 1; phenol index, naphthalene, and fluoranthene at Spot 2; and phenol index, naphthalene, chrysene, and total PAHs at Spot 3).

The successful use of a microbial consortium for PAH degradation was reported by other researchers. Fulekar [25] tested a bacterial consortium against naphthalene and anthracene, and their results showed a 98.4% reduction of naphthalene in 10 days and an 85.6% reduction of anthracene in 14 days. Both PAHs had an initial concentration equal to 500 mg/L. However, these results were carried out in vitro in a highly controllable environment. Moreover, research conducted by Jacques et al. [2] was conducted in soil under controlled conditions. They tested a consortium against anthracene, phenanthrene, and pyrene. After 90 days, the consortium mineralized 99% of added PAHs, in comparison to the soil not inoculated with the consortium (autochthonous microorganisms mineralized 5%, 34%, and 2% of added PAHs, respectively).

It has to be emphasized that our approach also has certain limitations. The collaboration with the industrial partner, and a restricted budget, limited the experimental plan, including a finite number of analyzed samples and their properties, technical repetitions, and additional controls. For that reason, the studies did not allow us to investigate by-products that can arise in the bioremediation process. Such data would strengthen the certainty of the obtained results. Nevertheless, the analyses were conducted in an accredited laboratory (NLF), improving the reliability of our outcome and the possibility that the selected consortium could have a positive impact on the bioremediation process. Additionally, another limitation could be a shortage of nutrients (e.g., nitrogen, phosphorus). Breedveld and Sparrevik [79] pointed out that this limitation can be particularly significant for 4-ring PAHs that require a specific amount of nutrients, as opposed to 2- or 3-ring PAHs that are degraded more easily.

## 4. Materials and Methods

### 4.1. Soil Samples and Contamination Measurements

The area is characterized by a slight decline towards surface waters, from which it is isolated by a concrete wharf. Groundwater in this area is shallow (about 1.7 m below ground level), and its level is strongly related to the level of the nearby surface waters. Changes in the groundwater level and the terrain are conducive to the horizontal and vertical displacement of pollutants in the ground, which makes it problematic to determine which factors, and with what force, influence the variable content of hydrocarbons in the soil (hydrocarbon displacement in the study area or the hydrocarbon distribution stimulated by microbial potential). The soil samples which were analyzed in this research originated from the harbor area where embankment ground (industrial soil) was deposited at the beginning of twentieth century to enable industrial activity. The thickness of the embankment layer ranges from 1.8 to 2.8 m and the composition of it consists of slag with humus admixtures and sand (loose, fine, and clayey sands), and brick debris. Organic silt and peat are compressed under the embankment. The analyzed soil has been characterized by chemical determinations: pH (pH-H_2_O) and pH in KCl (pH-KCl) according to PN-ISO 10390:1997 using glass electrode in a 1:5 (*v*/*v*) suspension of the soil in water (pH-H_2_O) or in solution of potassium chloride 1 mol/L (pH-KCl); organic carbon and hummus content in air-dry mass based on Tyurin′s method (hummus content was calculated by multiplying carbon content by the Van Bemmelen′s factor (1.724)); P_2_O_5_ content using extraction in calcium lactate and UV-VIS spectroscopy; total nitrogen was measured based on Kjeldahl′s method. Any determination on soil samples referring to air-dry mass pertains to a fine-earth fraction, where grain size is smaller than 2 mm. The obtained values are as follows: pH 7.9 (in KCl) and 8.0 (in H_2_O), organic carbon content of 2.90% air- dry mass and 5.00% of hummus air-dry mass, P_2_O_5_ content of 12.3 mg/100 g of soil and total nitrogen of 0.08%. The area is characterized by a slight slope towards surface waters, from which it is insulated with a concrete quay. In this area, groundwater is shallow (approx. 1.7 m below ground) and its level is strongly related to the level of nearby surface waters. Analyzed soil is contaminated with phenol and polycyclic aromatic hydrocarbons that relate to roofing tar production in this area since 1920. The industrial activity was formally completed in 1997. However, waste leftover after production has not been removed and is a source of contamination. The concentration of PAHs and phenol started to be monitored in 2015. At this time 13 of 14 points were contaminated (the average total PAHs of 14 points was 762.9 mg/kg). During the next three years the concentrations of these substances lowered to the acceptable level of 10 of 13 points (average of the self-cleansed points was 37.24 mg/kg). However, 3 points remained strongly contaminated (average of 3 most contaminated points was 1288 mg/kg in 2015 and 2032 mg/kg in 2018). These points were subjected to the biodegradation process described in paragraph 4.5. Moreover, the concentrations of heavy metals in the soil were measured in 2015 and the results are shown in the Supplementary Materials (Table S1).

**Figure 1:**
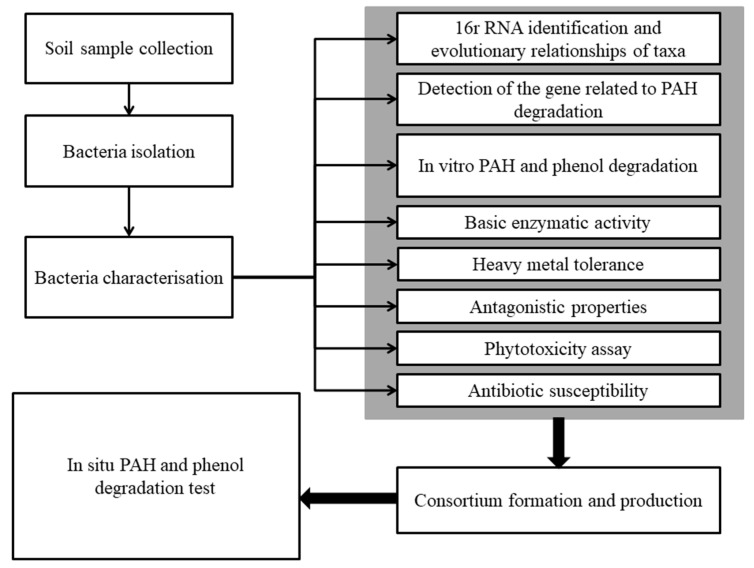
The design of the study.

The design of the study is presented in Figure 1. The soil sampling was carried out at a depth of 0 to 2 m according to the standard PN-ISO 10381-5:2009 (soil quality—sampling) from 14 measuring points divided into 3 layers (0.00–0.25 m, 0.25–1.00 m, and 1.00–2.00 m), resulting in 42 soil samples. 

The soil pollution assessment was carried out in accordance with the Regulation of the Minister of the Environment of 9 September 2002 on soil quality standards and ground quality standards (*Journal of Laws of the Republic of Poland* 2002 No. 165, item 1359). Analysis of the hydrocarbon concentration in the soil was carried out in accordance with PN-ISO 13877:2004 (soil quality—determination of polycyclic aromatic hydrocarbons) in the National Laboratory for Feedingstuffs (NLF) (accreditation certificate nr AB868 by Polish Centre for Accreditation). The contamination was assessed both before the application of the microbiological consortium in May 2019 and after in situ treatment with isolated bacteria in October 2019 and in June 2020.

### 4.2. Bacteria Isolation and Characterization

Bacteria isolation was based on a procedure proposed by John et al. [11] with minor modifications. The medium used for isolation was Mineral Medium (2.13 g Na_2_HPO_4_, 1.3 g KH_2_PO_4_, 0.5 g NH_4_Cl, and 0.2 g MgSO_4_ × 7H_2_O), trace elements solution 1 mL (FeSO_4_ × 7H_2_O-0.1 g, MnCl_4_ × 7H_2_O-0.1 g, ZnSO_4_ × 7H_2_O-0.1 g, and distilled water—100 mL), and 15 g agar per 1 L. Two grams of each soil sample were collected and suspended in a PBS buffer in ratio 1:9 (*w*/*v*), shook well for 10 min, and left to sediment, and the supernatant was used for further tests. The concentrations of PAHs equal to 0.125%, 0.250%, 0.500%, and 1.000% *w*/*v* were prepared by dissolving naphthalene (N), phenanthrene (Ph), anthracene (A), fluoranthene (F), chrysene (C), phenol (P), and a mix of all PAHs and phenol ((PAH + P) in proportions 3:2.5:1:2:1:2 (*v*/*v*)—related to the level of contamination measured before the intervention) in acetone. Then, the mineral medium was prepared on Petri dishes, and a layer (100 µL) of individual PAH solution, P, and PAH + P was spread on top of the solid medium. After evaporation of the acetone, 100 µL of the supernatant sample was plated on the agar surface and incubated for 7 days at room temperature. Afterward, colonies expressing different morphologies were chosen for further selection through the spot-tests that were carried out according to those by John et al. [11]. In the spot assay, only 0.5% solution of individual PAH, P, and PAH + P was layered on the surface of the solid mineral medium. Afterward, spots (3 µL) of each of the previously selected strain suspensions (0.5 McFarland) were applied and incubated for 7 days at room temperature. Following this, the ability of the strains to grow on the medium enriched with particular PAH, P, and PAH + P was assessed by the scoring method. The growth on each medium scored one point, and the strains with the highest score were further investigated. For this purpose, a method proposed by Fulekar [50] was used with the following modifications to eliminate the strains using agar as a carbon source. PAH + P was dissolved in acetone in proportion 3:2.5:1:2:1:2 (*v*/*v*) to obtain the concentration of 0.5% *w*/*v*. A quantity of 200 µL of the mixture was added to a sterile Erlenmeyer flask (100 mL) and was left until the solvent evaporated. After that, 19 mL of Mineral Medium and 1 mL of the individual bacteria culture in PBS buffer (0.5 McFarland standard, MF, and 1.2 × 10^6^–1.5 × 10^8^ CFU/mL) were added. The cultures were incubated for 4 days at room temperature and agitated at 250 rpm in a Shaker-Incubator ES-20/60 (BioSan, Latvia). Samples were collected daily from each of the flasks, ten-fold diluted in PBS buffer, and plated on Trypticase Soy Agar (Oxoid, UK). In the final step, those strains with the ability to grow in tested conditions were chosen for consortium development. Identification, taxonomic affiliation, evolutionary relationships, and additional physiological characteristics, including basic enzymatic activity, tolerance to heavy metals, antagonistic interactions with other isolates, phytotoxicity on *Lepidium sativum*, and antimicrobial susceptibility, were tested to obtain auxiliary data allowing the compatibility assessment of the created bacterial consortium. These data are shown in the Supplementary Material.

### 4.3. Detection of PAH-Degradation-Related Gene

The potential of the strains to degrade PAHs was also controlled by the detection of *pahE* (PAH hydratase-aldolase-encoding gene), as proposed Liang et al. [25]. The used primer pairs included pahE1F (TGCGGCGGGTGTNAAYGGNAT) and pahE1R (CCTGAGGAATCTCGGACATYTSTGCCCARAA), pahE2F (AGCATGGGAACKYTKGGNGA) and pahE2R (TTTGGCGGTVACVACYTG), pahE3F (GACGGCGTSGACGGVATCAT) and pahE3R (TCAGGGTTGTCRTARAKSA), and pahE4F (TGGTGCGYGAYGGBGYCGA) and pahE4R (GGCGTGCGGGTTSTSRTARAYCA). PCR was carried out in 25 µL reaction mixture: 6.25 µL RT PCR Mix SYBR^®^A, A&A Biotechnology, Poland; 0.2 µM of each primer forward and reverse, 4.75 µL molecular DNase-free water, and 1.0 µL of bacterial genomic DNA. The reaction was carried out with the following settings: initial denaturation at 94 °C for 5 min, followed by 35 cycles of 94 °C for 30 s, 52 to 57 °C (52 °C for *pahE1*, 57 °C for *pahE2*, and 54 °C for *pahE3* and *pahE4*) for 45 s, and 72 °C for 30 s, with a final extension at 72 °C for 10 min in thermocycler CFX Connect Real-Time PCR Detection System, Life Science, Bio-Rad^®^.

### 4.4. Hydrocarbons Degradation In Vitro Test

The degradation of PAHs and phenol was analyzed based on the method proposed by Fulekar [66]. Each PAH or phenol was dissolved in acetone to obtain a concentration of 0.5% (*w*/*v*). After that, the solutions were mixed in proportion 3:2.5:1:2:1:2 (*v*/*v*) corresponding to the contamination ratio in the soil of origin. The final content of each substance equaled: naphthalene—2.6 mg/L; phenanthrene—2.2 mg/L; anthracene—0.9 mg/L; fluoranthene—1.7 mg/L; chrysene 0.9 mg/L; and phenol—1.7 mg/L. In the tested samples, 2 mL of the PAH mixture was added to a sterile bottle (1000 mL) and left until the evaporation of the solvent. After that, 950 mL of Mineral Medium and 50 mL of bacterial consortium containing equal proportions of each bacterium (0.5 MF) were added. Control samples did not include the consortium of microorganisms. One control and one tested sample were collected for the analysis after 1 day and after 8 days of incubation at room temperature and with continuous mixing at 250 rpm (Shaker-Incubator ES-20, BioSan, Latvia).

The degradation rate was evaluated using the spectrophotometric method according to PN-ISO 6439:1994 (for phenol index) and high-performance liquid chromatography (HPLC) according to PN-EN ISO 17993:2005 (water quality—determination of 15 polycyclic aromatic hydrocarbons (PAHs) in water by HPLC with fluorescence detection after liquid–liquid extraction). The analyses were carried out by NLF.

### 4.5. Consortium Development and Application to Soil

The usefulness of the consortium of microorganisms was evaluated in field conditions. Selected bacteria were separately cultivated until the stationary phase (7.0 MF) was reached. To confirm the viability and number of each bacteria, the standard plate count assay was performed. The titer of bacteria varied from 1 × 10^7^–1 × 10^9^ CFU/mL for MF = 7. A wide range of titres was due to the different morphologies of the used bacterial cells. The viability of microorganisms was confirmed by a standard plate count assay. In the next step, single-species cultures were mixed in equal proportions, which resulted in 80 L of co-culture. Afterward, the consortium was immediately transported to the test field and diluted on-site in water to obtain 0.5 MF (1.2 × 10^6^–1.5 × 10^8^ CFU/mL). The final volume of the consortium equaled 1000 L. The suspension was equally spread in the 3 most polluted spots on the contaminated area (2 m^2^ each) on the surface of the soil and into deeper layers by boreholes. Before the application, the soil was richly watered to improve soil sorption capacity. Other agricultural procedures or soil enrichment were not conducted. The procedure was repeated in one-week intervals during the first three weeks of the experiment. The reasoning behind such planning was to include possible weather changes in the area and the available volume of the containers for our consortium. Furthermore, weekly repetition of the inoculations in the soil was conducted to achieve the growth and survival of the bacteria in the contaminated soil [80]. After five (October 2019) and thirteen months (June 2020), the concentration of PAHs in those three spots was measured by NLF. The soil sampling was carried out at a depth of 0 to 2 m according to the standard PN-ISO 10381-5:2009. Each of the 3 points was divided into three layers (0.00–0.25 m, 0.25–1.00 m, and 1.00–2.00 m). The mean values from the three spots obtained before and after intervention were compared.

## 5. Conclusions

This research can be regarded as a case study for the collaboration between science and an industrial partner. Autochthonic microorganisms isolated from contaminated soil have been tested in a series of experiments which have shown the potential to remove PAHs, in particular, as a developed consortium. The methods elucidating the physiological properties of the isolates have confirmed the utility of the consortium for the intended application. The used approach could be implemented by other groups working on bioremediation techniques aiming at elevating the numbers of potent autochthonic bacteria in cleanup processes. The gathered experimental data suggest that the application of autochthons has had a positive impact on the bioremediation process. However, it has to be emphasized that despite the application of the microbiological consortium, the concentration on most selected PAHs was still above the ministerial threshold (50 mg/kg) after 13 months and at the end of the project. Nevertheless, our approach was enough to achieve the acceptable level of degradation of naphthalene, whereas the concentration of other PAHs was reduced in respect to the state before the consortium was applied.

## Figures and Tables

**Table 1:** Presence of genes related to PAHs decomposition in isolated strains: (+) presence of the gene, (-) absence of the gene.

Strain	Strain Genera	Gene			
		*pahE1*	*pahE2*	*pahE3*	*pahE4*
11	*Microbacterium* sp.	-	-	-	+
17	*Pseudomonas* sp.	+	-	+	+
19	*Streptomyces* sp.	+	-	-	-
20	*Achromobacter* sp.	-	-	-	+
22	*Cupriavidus* sp.	+	+	+	+
24	*Rhodococcus* sp.	-	-	+	+
26	*Arthrobacter* sp.	-	-	+	+
27	*Arthrobacter* sp.	-	-	+	+
29	*Arthrobacter* sp.	+	+	+	+
36	*Pseudomonas* sp.	+	+	+	+
38	*Pseudomonas* sp.	+	+	+	+
40	*Rhodococcus* sp.	+	-	+	+
41	*Pseudomonas* sp.	+	-	+	+
44	*Rhodococcus* sp.	+	-	+	+

**Table 2:** PAHs and phenol content in the samples after: (a) 1 day, (b) 8 days of incubation as a percentage of the calculated initial concentration of PAHs and phenol.

Hydrocarbon	Substance Content in Comparison to the Initial Calculated Content [%]			
	1 Day of Incubation		8 Days of Incubation	
	Control Sample (without Consortium)	Tested Sample (Added Consortium)	Control Sample (without Consortium)	Tested Sample (Added Consortium)
Phenol	73.06	62.82	65.29	0.29
Naphthalene	19.23	15.00	10.00	0.04
Phenanthrene	21.36	8.64	17.27	1.73
Anthracene	8.33	2.33	4.89	0.78
Fluoranthene	27.65	2.53	13.53	1.82
Chrysene	4.44	0.52	1.33	0.20
Total PAHs	18.39	7.47	10.69	0.90

**Table 3:** Hydrocarbon concentration in soil (3 spots).

Hydrocarbon	Acceptable Concentration * [mg/kg]	Concentration [mg/kg] in Soil (0–2 m below Ground Level)								
		05/2019			10/2019			06/2020		
		Spot 1	Spot 2	Spot 3	Spot 1	Spot 2	Spot 3	Spot 1	Spot 2	Spot 3
Phenol index	50	23.2	17.8	12.5	1.7	3.5	0.8	3.8	0.28	0.17
Naphthalene	50	407.0	108.0	135.0	353.0	129.0	5.5	74.7	13.6	17.0
Phenanthrene	50	736.0	144.0	1073.0	500.0	121.0	39.0	698.1	154.2	212.3
Anthracene	50	692.0	144.0	675.0	670.0	119.0	33.7	431.2	102.2	110.4
Fluoranthene	50	503.0	175.0	76.0	375.0	500.0	43.5	455.1	18.5	141.6
Chrysene	50	148.0	68.0	136.0	127.0	40.7	16.6	138.4	58.1	27.3
Total PAHs	250	2635.0	724.0	2289.0	2344.0	1265.0	160.0	977.4	349.0	242.0

* According to the Regulation of the Minister of the Environment of 9 September 2002 on soil quality standards and ground quality standards (*Journal of Laws of the Republic of Poland* 2002 No. 165, item 1359).

**Table 4:** Percentage of the hydrocarbons in comparison to initial values before consortium application.

Hydrocarbon	Percentage of the 05/2019 Value [%]					
	10/2019			06/2020		
	Spot 1	Spot 2	Spot 3	Spot 1	Spot 2	Spot 3
Phenol index	7.3	19.7	6.7	16.3	1.57	1.33
Naphthalene	86.7	119.4	4.1	18.4	12.57	12.6
Phenanthrene	67.9	84.0	3.6	94.9	107.1	19.8
Anthracene	96.8	82.6	5.0	62.3	71.0	16.4
Fluoranthene	74.6	285.7	57.2	90.5	10.6	186.4
Chrysene	85.8	59.9	12.2	93.5	85.48	20.1
Total PAHs	88.9	174.7	7.0	37.1	48.21	10.6

## Data Availability

16S rRNA sequences were stored in GenBank database under the following accession numbers: MT755842-MT755855 (forward) and MT755859-MT755872 (reverse).

## Supplementary Materials

The following are available online at https://www.mdpi.com/article/10.3390/ijms222413469/s1.
