# Supplementary material for: Development of an Autochthonous Microbial Consortium for Enhanced Bioremediation of PAH-Contaminated Soil
Source: Int J Mol Sci. 2021 Dec 15;22(24):13469. doi: 10.3390/ijms222413469 (PMC8708151; doi:10.3390/ijms222413469)
Supplement: Supplementary file 1 [file ijms-22-13469-s001.zip › Roszak_et_al_suppl_revised.pdf]

Supplementary material

Development of autochthonous microbial consortium for enhanced bioremediation of PAH-contaminated soil

Marta Roszak[1,2†], Joanna Jabłońska[1,3†], Xymena Stachurska[1], Kamila Dubrowska[1,3], Justyna Kajdanowicz[1], Marta Gołębiewska[1], Anna Kiepas-Kokot[4], Beata Osińska[5], Adrian Augustyniak[1,2,6*], Jolanta Karakulska[1]

[1] West Pomeranian University of Technology, Szczecin, Faculty of Biotechnology and Animal Husbandry, Department of Microbiology and Biotechnology, Al. Piastów 45, 70-311 Szczecin, Poland

[2] Pomeranian Medical University in Szczecin, Chair of Microbiology, Immunology, and Laboratory Medicine, Department of Laboratory Medicine, Al. Powstańców Wielkopolskich 72, 70-111 Szczecin, Poland

[3] West Pomeranian University of Technology, Szczecin, Faculty of Chemical Technology and Engineering, Department of Chemical Engineering, Al. Piastów 42, 71-065 Szczecin, Poland

[4] West Pomeranian University of Technology, Szczecin, Faculty of Environmental Management and Agriculture, Department of Environmental Management, ul. Słowackiego 17, 71-434 Szczecin, Poland

[5] Research Institute of Animal Production PIB Kołbacz Sp. z o.o., Warcisława Street 1, 74-106 Stare Czarnowo, Poland

[6] Technische Universität Berlin, Chair of Building Materials and Construction Chemistry, Gustav-Meyer-Allee 25, 13355 Berlin, Germany

***Corresponding author:** Adrian Augustyniak, West Pomeranian University of Technology in Szczecin, Faculty of Chemical Technology and Engineering, Department of Chemical Engineering, Al. Piastów 42, 71-065 Szczecin, Poland; e-mail: adrian.augustyniak@zut.edu.pl

Materials and methods

Identification and taxonomic affiliation

DNA extraction.

DNA was isolated from 14 strains using the Genomic Mini kit (A&A Biotechnology, Poland).

Amplification of 16S rRNA gene

The fragments above 1000 bp of 16S rRNA gene were amplified using primers 8f (5'-AGAGTTTGATCATGGCTCAG) and 1492R (5’-CGGTTACCTTGTTACGACTT) previously described by Xiang et al. (2005). The PCR was performed in a final volume of 12.5 μL: 6.25 μL of MasterMix (A&A Biotechnology, Poland), 0.25 μL of each primer (10 mM), 4.75 μL of endonuclease-free water and 1 μL of DNA. The PCR conditions were optimized as follows: initial denaturation at 95°C for 15 minutes, 40 cycles: DNA denaturation at 95 °C for 45 s, annealing of primers for 45 s at 55°C, extension at 72 °C for 45 s; final extension at 72 °C for 5 min.

16S rRNA gene sequencing

Sequencing was outsourced to Nexbio company (Lublin, Poland). Sequencing analysis was carried out using the BLAST program, based on the GenBank nucleotide database (on 14.07.2020) at the National Centre for Biotechnology Information (NCBI) website. Sequences were stored in GenBank database under following accession numbers: MT755842-MT755855 (forward) and MT755859-MT755872 (reverse).

Evolutionary relationships of taxa

The evolutionary history was inferred using the Neighbor-Joining method [2]. The bootstrap consensus tree inferred from 1000 replicates [3] is taken to represent the evolutionary history of the taxa analysed [3]. Branches corresponding to partitions reproduced in less than 50% bootstrap replicates are collapsed. The percentage of replicate trees in which the associated taxa clustered together in the bootstrap test (1000 replicates) are shown next to the branches [3]. The evolutionary distances were computed using the Maximum Composite Likelihood method (Tamura et al., 2004) and are in the units of the number of base substitutions per site. This analysis involved 14 nucleotide sequences. All ambiguous positions were removed for each sequence pair (pairwise deletion option). There were a total of 1100 positions in the final dataset. Evolutionary analyses were conducted in MEGA X [5].

Basic enzymatic activity

Basic enzymatic activity of isolates was studied with the use of API ZYM kit (Biomerieux, France) according to user’s manual. API ZYM assay was used to study enzymes involved in carbon, nitrogen and phosphorus cycles also by other authors [6].

Tolerance to heavy metals

The soil was assessed in 2015 in context of possible heavy metal contamination. For measurement content of Zn, Cd, Pb, Cu and Ni method described in PN-ISO 11047:2001 was used (soil extraction with aqua regia, flame and electrothermal atomic absorption spectroscopy methods). For Hg content measurement method based

on AMA analyser (Atomic Absorption Spectroscopy) and amalgamation technique. The mean values of 3 tested areas are presented in Table S1.

Table S1. The mean value with standard deviation of heavy metal concentration (presented in mg/kg of soil)

Depth of the sampling [m]	Zn	Cd	Hg	Pb	Cu	Ni
0-0.5	200.33±31.21	0.24±0.16	0.15±0.07	51.4±34.45	19.93±1.03	8.60±0.58
0.5-2.	75.03±47.24	0.36±0.09	0.16±0.08	132.90±28.69	6.98±2.31	4.00±1.36

Obtained values were not exceeding the values allowed by the Polish law. However, heavy metal contamination is often associated with PAHs contamination, hence the analysis of tolerance to heavy metals was carried out to additionally assess the potential of the isolated strains. Seven heavy metals (Zn, Cu, Co, Ni, Cd, Hg, Pb) were selected to carry out tolerance tests. Heavy metal water solutions were prepared in concentrations of 50 mM of $ZnSO_4 \times 7H_2O$, $CuSO_4 \times 5H_2O$, $NiCl_2 \times 6H_2O$, $CoCl_2 \times 6H_2O$, $CdSO_4$ and 30 mM of $HgCl_2$ and $PbCl_2$ (due to lower solubility in water). In the next step, solutions were filtered using PES syringe filters with 0.22 µm pores. Afterwards, 100 µL of each solution was added onto 96-well microtitrate plates and the series of twofold dilutions was prepared in order to obtain the following range of concentrations: 25 mM – 0.20 mM (for Zn, Cu, Cd), 12.5 – 0.1 mM (Co and Ni) 7.5 mM – 0.06 mM (Pb) and 3.75 mM – 0.03 mM (Hg). Then, 100 µL of selected bacterial cultures (at 0.5 MF) in TSB medium were added onto the wells. The cultures were incubated at room temperature for 48h. The optical density (OD_{600nm}) was measured on Infinite 200 PRO NanoQuant spectrophotometer (Tecan, Männedorf, Switzerland). TSB with distilled water was used as the blank sample and bacterial culture with metal-free water was applied as growth control. The experiment was conducted in triplicate. Maximum tolerable concentration (MTC) of metals, defined as the highest concentration of heavy metal with the increase of turbidity of bacterium culture after 48 hours, was determined according to OD_{600nm} measurements.

Antagonistic assay

Antagonistic activity was tested according to the method proposed by Jacobsen et al. (1999) with minor modifications. Each strain was inoculated and incubated in a Muller-Hinton Broth (BioMaxima, Poland) for 24 hours at the room temperature. After incubation, each of the bacterial inoculum (0.5 MF) was spread on Muller-Hinton Agar (BioMaxima, Poland). The plates were then incubated agar down at room temperature for approximately 1h, for the inocula to settle in agar. Afterwards, the spot-test was carried out. 2 µL of the previously prepared liquid cultures (0.5 MF) of all other strains were applied onto Muller-Hinton Agar plates and incubated for 24 hours at room temperature.

Positive result was obtained if the inhibition zone around the bacterial colony was equal or greater than 1 mm in diameter. The lack of visible growth inhibition was marked as a negative result (Jacobsen et al., 1999).

Phytotoxicity on *Lepidium sativum L.* model

Lepidium sativum root and shoot inhibition tests were based on ISO 11269-1:2012 standard and carried out according to Favier et al. (2019) with following modifications. *L. sativum* seeds were preincubated on sterile lignin moistened with water for 24 hours, until the sprouting process started. Afterwards, sterile garden soil was placed on Petri dishes and the suspensions of each analysed bacteria (10 mL; 0.5 MF) were mixed with the soil. 25 sprouting seeds were then seeded in each plate. Tap water was used for the control test. The length of roots and shoots was measured after 5 days of incubation at the room temperature.

The results were analysed statistically with Statistica 13.3 TIBCO Software Inc. (StatSoft Inc., Tulsa, OK, USA). One-way ANOVA was performed with Tukey's post-hoc test was used to compare the results. Differences were considered significant at $p \leq 0.05$.

Antimicrobial susceptibility testing (AST)

The susceptibility of bacterial strains to antimicrobial agents was assessed by the disk diffusion method, in accordance with the standards of The European Committee on Antimicrobial Susceptibility Testing (EUCAST, 2020). *Pseudomonas aeruginosa* ATCC® 27853™ and *S. aureus* ATCC® 25923™ were used as control strains. The antibiotic disks (Biomaxima, Poland) used in this phase of the project are indicated in Table 3 and Table 4 along with doses and abbreviations.

Several isolated genera are not included in EUCAST recommendations. Therefore, recommendations for phylogenetically related bacteria were used in these cases. The antimicrobial susceptibility of *Achromobacter* and *Cupriavidus* strains was interpreted according to EUCAST breakpoints for *Pseudomonas* genus, while the antibiotic sensitivity of *Rhodococcus*, *Arthrobacter*, *Microbacterium* and *Streptomyces* strains was compared with EUCAST breakpoint tables for *Corynebacterium* genus [9].

Results

Identification

Table S2. 16s rRNA identification of the strains

Strain number	Genus	Phylogenetic tree
20	*Achromobacter* sp.	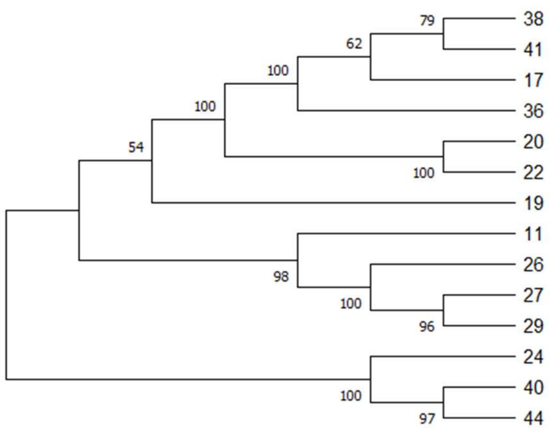
26	*Arthrobacter* sp.	
27	*Arthrobacter* sp.	
29	*Arthrobacter* sp.	
11	*Microbacterium* sp.	
17	*Pseudomonas* sp.	
36	*Pseudomonas* sp.	
38	*Pseudomonas* sp.	
41	*Pseudomonas* sp.	
22	*Cupriavidus* sp.	
24	*Rhodococcus* sp.	
40	*Rhodococcus* sp.	
44	*Rhodococcus* sp.	
19	*Streptomyces* sp.	

Basic enzymatic activity

Table S3. The results of the enzymatic activity assay

Enzyme	Enzyme function	Strain													
		11	17	19	20	22	24	26	27	29	36	38	40	41	44
Alkaline phosphatase	dephosphorysation (pH>7)	+	+	+	+	+	+	+	+	+	+	+	+	+	+
Esterase (C 4)	splits esters into an acid and an alcohol	+	+	+	+	+	+	+	+	+	+	+	+	+	+
Esterase Lipase (C 8)	hydrolysis of glycerolesters with short chains (<C8)	+	+	+	+	+	+	+	-	+	+	+	+	+	+
Lipase (C 14)	hydrolysis of lipids (<C14)	-	-	+	-	-	+	-	+	+	+	-	+	-	-
Leucine arylamidase	hydrolysis of an N-terminal L-leucine from peptide substrates	+	+	+	+	+	+	+	+	+	+	+	+	+	+
Valine acrylamidase	hydrolysis of an N-terminal L-valine from peptide substrates	+	-	+	+	+	+	+	+	+	+	-	+	-	+
Cystine acrylamidase	hydrolysis of an N-terminal L-cystine from peptide substrates	+	-	+	-	-	+	+	+	+	+	-	+	-	+
Trypsin	hydrolysis of peptide bonds in places where the carbonyl groups belong to arginine or lysine	+	-	-	-	+	+	+	+	+	+	-	+	-	+
α -chymotrypsin	hydrolysis of an N-terminal L-tryptophan, tyrosine, phenylalanine or leucine from peptide substrates	-	-	+	-	+	+	-	-	+	-	-	+	-	+
Acid phosphatase	dephosphorysation (pH<7)	+	+	+	+	+	+	+	+	+	+	+	+	+	+
Naphtol-AS-BI-phosphohydrolase	hydrolysis of naphtol AS-BI phosphate	+	+	+	+	+	+	+	+	+	+	+	+	+	+
α- galactosidase	hydrolyzes the α-glycosidic bond formed between a galactose and its organic moieties	+	-	-	-	-	-	+	+	+	-	-	-	-	-

β-galactosidase	hydrolysis the β-glycosidic bond formed between a galactose and its organic moiety	+	-	+	-	-	+	+	+	+	-	-	+	-	+
β - glucoronidase	hydrolysis of β-D-glucuronic acid	-	-	-	-	-	-	+	+	+	-	-	-	-	-
α -glucosidase	hydrolysis of terminal non-reducing 1,4-linked α-glucose to release α-glucose	+	-	-	-	-	+	+	+	+	-	-	+	-	+
β - glucosidase	hydrolysis of terminal non-reducing 1,4-linked β-glucose to release β-glucose	+	-	+	-	-	+	+	+	+	-	-	+	-	+
N-acetyl- β-glucosaminidase	degradation of glycoproteins, glycolipids and glycosaminoglycans	+	-	+	-	-	-	-	-	-	-	-	-	-	-
α - mannosidase	cleavage of the α form of mannose	+	-	+	-	-	-	+	+	+	-	-	-	-	-
α -fucosidase	hydrolysis of the α-fucoside to fucose and alcohol	-	-	-	-	-	-	-	-	-	-	-	-	-	-

Tolerance to heavy metals

The results have shown that all strains are able to survive in concentrations higher than 0.78 mM for Zn and Ni, 0.20 mM for Co, 1.56 mM for Cu, and 0.47 mM for Pb. The highest reported MTC for zinc equalled 6.25 mM, for nickel – 3.13 mM, for cobalt – 0.78 mM, for copper – 6.25, for cadmium 6.25 and for lead – 15 mM. None of the strains were able to tolerate mercury and 2 isolates could not grow in the presence of cadmium. Isolated pseudomonads (17 and 38) achieved the highest MTC values in 4 and 5 out of 6 metals tested (excluding mercury), respectively. Tolerance to heavy metals among isolates as shown in Table S4.

Table S4. Obtained MTCs of the tested heavy metals on the isolated strains

Strain number	Genus	MTC obtained for each heavy metal [mM]					
		Zn	Ni	Co	Cu	Cd	Pb
20	*Achromobacter* sp.	6.25	0.78	0.20	3.13	6.25	3.75
26	*Arthrobacter* sp.	3.13	0.78	0.20	3.13	1.56	3.75
27	*Arthrobacter* sp.	3.13	0.78	0.20	3.13	3.13	1.88
29	*Arthrobacter* sp.	6.25	1.56	0.20	3.13	6.25	3.75
11	*Microbacterium* sp.	3.13	0.78	0.20	3.13	NA	0.23
17	*Pseudomonas* sp.	6.25	0.78	0.39	3.13	6.25	7.50
36	*Pseudomonas* sp.	3.13	0.78	0.20	6.25	0.78	3.75
38	*Pseudomonas* sp.	6.25	1.56	0.39	6.25	6.25	3.75
41	*Pseudomonas* sp.	0.78	0.39	0.20	3.13	0.39	3.75

22	*Cupriavidus* sp.	1.56	1.56	0.39	3.13	0.39	3.75
24	*Rhodococcus* sp.	3.13	0.78	0.20	3.13	0.78	3.75
40	*Rhodococcus* sp.	3.13	0.78	0.39	3.13	0.39	3.75
44	*Rhodococcus* sp.	6.25	0.78	0.20	1.56	NA	3.75
19	*Streptomyces* sp.	6.25	0.39	0.10	3.13	6.25	3.75

Antagonistic assay

9 of 14 analysed strains did not present antagonistic activity against subject strains, while 5 of them displayed inhibiting action (Table S5). 1 isolate (no. 38) showed its antagonistic activity against three of the examined strains (no. 26, 27 and 29), 3 isolates (no. 24, 40 and 44) – against two strains (no. 17 and 41), and 1 subject strain (no. 19) showed its inhibiting activity against one of the examined strains (no. 29) (Fig. S1).

Table S5. Antagonistic assay results

Strain spread on the surface of the medium/Strain tested	11	15	17	19	20	21	22	23	24	26	27	29	31	36	37	38	40	41	44
11		-	-	-	-	-	-	-	-	-	-	-	-	-	-	-	-	-	-
15	-		-	-	-	-	-	-	-	-	-	-	-	-	-	-	-	-	-
17	-	-		-	-	-	-	-	+	-	-	-	-	-	-	-	-	-	-
19	-	-	-		-	-	-	-	-	-	-	+	-	-	-	-	-	-	-
20	-	-	-	-		-	-	-	-	-	-	-	-	-	-	-	-	-	-
21	-	-	-	-	-		-	-	-	-	-	-	-	-	-	-	-	-	-
22	-	-	-	-	-	-		-	-	-	-	-	-	-	-	-	-	-	-
23	-	-	-	-	-	-	-		-	-	-	-	-	-	-	-	-	-	-
24	-	-	+	-	-	-	-	-		-	-	-	-	-	-	-	-	+	-
26	-	-	-	-	-	-	-	-	-		-	-	-	-	-	-	-	-	-
27	-	-	-	-	-	-	-	-	-	-		-	-	-	-	-	-	-	-
29	-	-	-	-	-	-	-	+	-	-	-		-	-	-	-	-	-	-
31	-	-	-	-	-	-	-	-	-	-	-	-		-	-	-	-	-	-
36	-	-	-	-	-	-	-	-	-	-	-	-	-		-	-	-	-	-
37	-	-	-	-	-	-	-	-	-	-	-	-	-	-		-	-	-	-
38	-	-	-	-	-	-	-	-	-	+	+	+	-	-	-		-	-	-
40	-	-	+	-	-	-	-	-	-	-	-	-	-	-	-	-		+	-
41	-	-	-	-	-	-	-	-	-	-	-	-	-	-	-	-	-		-
44	-	-	+	-	-	-	-	-	-	-	-	-	-	-	-	-	-	+	

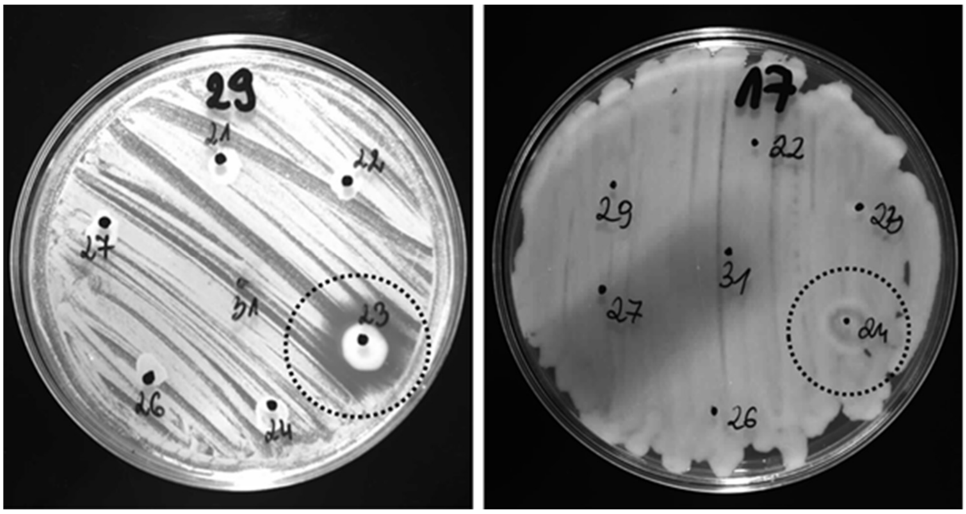

Figure S1. Antagonistic activity of the tested bacterial strains. Positive results were identified as inhibition zones (bacterial lawn clearance) greater than or equal to 1 mm diameter around bacterial colony. Examples of strains growth inhibition (no. 29 and 17) were marked with a dotted circle

Phytotoxicity on *Lepidium sativum* model

Statistical analysis showed the occurrence of statistically significant differences between the length of the shoots in the control sample and the length of shoots in the tested samples ($p<0.05$). Strains no. 41 and 44 did not show a reducing effect on shoots growth compared to the control. Shoot samples paired with strains no. 11, 17, 19, 20, 22, 24, 26, 27, 29, 36, 38 and 40 were significantly ($p<0.05$) shorter, compared to the control (Figure S2). In addition, 7 strains (no. 17, 22, 24, 36, 38, 41 and 44) did not show a reducing effect on the growth of *L. sativum* roots. Seedlings paired with other strains (no. 11, 19, 20, 26, 27, 29 and 40) have been characterized with shorter roots compared to control samples.

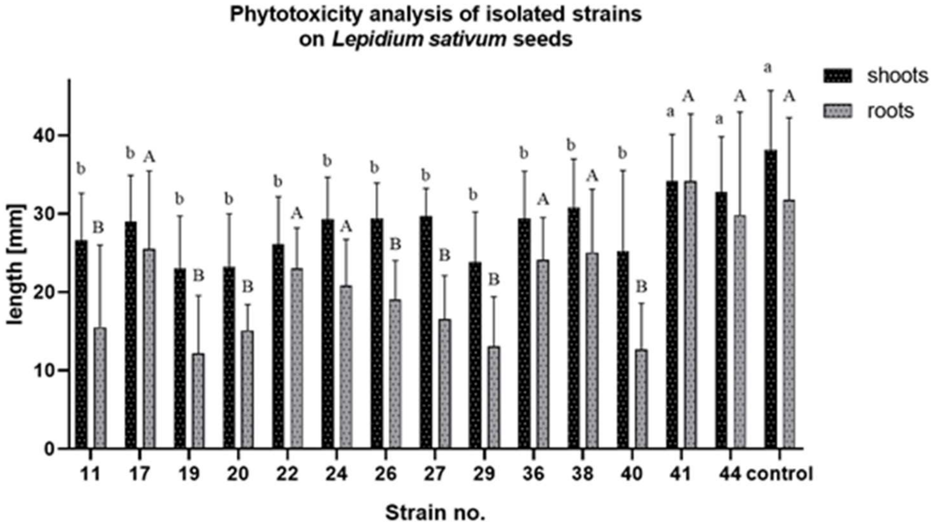

Figure S2. Phytotoxicity analysis of isolated strains on *L. sativum* seeds. Vertical bars indicate means ± SD. A, B (roots) and a, b (shoots) – means sharing the same superscript are not significantly different from each other at $p \leq 0.05$

Antibiotic resistance

Among 6 Gram-negative bacteria strains, four *Pseudomonas* spp. showed resistance to 2, 3, 4 and 5 antimicrobial agents out of 13 tested (Table S5). Resistance to ticarcillin and ticarcillin with clavulanic acid was noted in all of the *Pseudomonas* strains, while aztreonam resistance was demonstrated in 3 out of 4 strains. Resistance to piperacillin-tazobactam was detected in 1 strain, as well as resistance to ceftazidime and tobramycin. In turn, *Achromobacter* spp. strain showed resistance to 4 out of 13 antimicrobial agents tested (cefepime, aztreonam, amikacin, tobramycin), while *Cupriavidus* sp. showed resistance to ceftazidime (Table S6).

Among 8 Gram-positive bacteria, clindamycin resistance was noted in 6 strains, tetracycline resistance was demonstrated in 4 strains, ciprofloxacin resistance in 3 strains, rifampicin resistance in 2 strains and linezolid resistance in 1 strain (Table S6, Table S7).

The widest spectrum of resistance was exhibited by *Streptomyces* strain (strain No. 19), followed by *Arthrobacter* strain (No. 24). These strains were resistant to 4 and 3 out of 5 antimicrobial agents tested, respectively (Table S7).

Table S6. Heatmap of antibiotics resistance patterns for the Gram-negative strains. Red colour indicates resistant strain, yellow – intermediate and green – sensitive

Antimicrobial agent	Antimicrobial agent abbreviation	*Pseudomonas*				*Achromobacter*	*Cupriavidus*
		Strain no.					
		17	36	38	41	20	22
Piperacillin	PRL30						
Piperacillin-tazobactam	TZP36						
Ticarcillin	TC75						
Ticarcillin-clavulanic acid	TIM85						
Cefepime	FEP30						
Ceftazidime	CAZ10						
Imipenem	IPM10						
Meropenem	MEM10						

Aztreonam	ATM30						
Ciprofloxacin	CIP5						
Levofloxacin	LVX5						
Amikacin	AK30						
Tobramycin	TOB10						

Table S7. Heatmap of antibiotics resistance patterns for the Gram-positive strains. Red colour indicates resistant strain, yellow – intermediate and green – sensitive

Antimicrobial agent	Antimicrobial agent abbreviation	*Rhodococcus*			*Arthrobacter*			*Microbacterium*	*Streptomyces*
		Strain no.							
		26	27	29	24	40	44	11	19
Ciprofloxacin	CIP5								
Clindamycin	DA2								
Tetracycline	TE30								
Linezolid	LNZ10								
Rifampicin	RA5								

Hydrocarbons degradation test

Table. S8 The results of hydrocarbons degradation test (*The calculated content refers to the concentration of phenol that was chosen as a model substance)

Method of the analysis	Hydrocarbons concentration (µg/L)	Calculated initial content of hydrocarbons (µg/L)	Content of hydrocarbons after 1 day of incubation (µg/L)		Content of hydrocarbons after 8 day of incubation (µg/L)	
			Control sample	Tested sample	Control sample	Tested sample
Spectrophotometric method according to PN-ISO 6439:1994	Phenol index	1700*	1242	1068	1110	5
High-performance liquid chromatography (HPLC) according to PN-ENISO 17993:2005	Naphthalene	2600	500	390	260	1.1
	Phenanthrene	2200	470	190	380	38
	Anthracene	900	75	21	44	7
	Fluoranthene	1700	470	43	230	31
	Chrysene	900	40	4.7	12	1.8
	Total of PAHs	8300	1600	650	930	78

References

[1] Xiang S, Yao T, An L, Xu B, Wang J. 16S rRNA sequences and differences in bacteria isolated from

the Muztag Ata glacier at increasing depths. Appl Environ Microbiol 2005;71:4619–27. https://doi.org/10.1128/AEM.71.8.4619-4627.2005.

[2] Saitou N, Nei M. The neighbor-joining method: a new method for reconstructing phylogenetic trees. Mol Biol Evol 1987;4:406–25. https://doi.org/10.1093/oxfordjournals.molbev.a040454.

[3] Felsenstein J. Confidence Limits on Phylogenies: An Approach Using the Bootstrap. Evolution (N Y) 1985;39:783–91. https://doi.org/10.2307/2408678.

[4] Tamura K, Nei M, Kumar S. Prospects for inferring very large phylogenies by using the neighbor-joining method. Proc Natl Acad Sci U S A 2004;101:11030–5. https://doi.org/10.1073/pnas.0404206101.

[5] Kumar S, Stecher G, Li M, Knyaz C, Tamura K. MEGA X: Molecular Evolutionary Genetics Analysis across Computing Platforms. Mol Biol Evol 2018;35:1547–9. https://doi.org/10.1093/molbev/msy096.

[6] Das S, Gwon HS, Khan MI, Jeong ST, Kim PJ. Steel slag amendment impacts on soil microbial communities and activities of rice (Oryza sativa L.). Sci Rep 2020;10:1–11. https://doi.org/10.1038/s41598-020-63783-1.

[7] Jacobsen CN, Rosenfeldt Nielsen V, Hayford AE, Møller PL, Michaelsen KF, Paerregaard A, et al. Screening of probiotic activities of forty-seven strains of Lactobacillus spp. by in vitro techniques and evaluation of the colonization ability of five selected strains in humans. Appl Environ Microbiol 1999;65:4949–56.

[8] Favier L, Rusu L, Simion AI, Hlihor RM, Pacala ML, Augustyniak A. Efficient degradation of clofibric acid by heterogeneous photocatalytic oxidation process. Environ Eng Manag J 2019;18:1683–92. https://doi.org/10.30638/eemj.2019.158.

[9] European Committee on Antimicrobial Susceptibility Testing. Testing Breakpoint tables for interpretation of MICs and zone diameters 2020:0–77.
